# MiR-770-5p inhibits cisplatin chemoresistance in human ovarian cancer by targeting ERCC2

**DOI:** 10.18632/oncotarget.10736

**Published:** 2016-07-20

**Authors:** Henan Zhao, Xiaotang Yu, Yanfang Ding, Jinyao Zhao, Guang Wang, Xian Wu, Jiyong Jiang, Chun Peng, Gordon Zhuo Guo, Shiying Cui

**Affiliations:** ^1^ Dalian Medical University, Dalian China; ^2^ Obstetrics and Gynecology Hospital, Dalian China; ^3^ Department of Biology, York University, Toronto, Canada; ^4^ Department of Radiation Oncology, Indiana University School of Medicine, IN, USA

**Keywords:** ovarian cancer, cisplatin, chemoresisitance, miR-770-5p, ERCC2

## Abstract

In this study, we examined the role of the miRNA miR-770-5p in cisplatin chemotherapy resistance in ovarian cancer (OVC) patients. miR-770-5p expression was reduced in platinum-resistant patients. Using a 6.128-fold in expression as the cutoff value, miR-770-5p expression served as a prognostic biomarker and predicted the response to cisplatin treatment and survival among OVC patients. Overexpression of miR-770-5p *in vitro* reduced survival in chemoresistant cell lines after cisplatin treatment. ERCC2, a target gene of miR-770-5p that participates in the NER system, was negatively regulated by miR-770-5p. siRNA-mediated silencing of ERCC2 reversed the inhibition of apoptosis resulting from miR-770-5p downreglation in A2780S cells. A comet assay confirmed that this restoration of cisplatin chemosensitivity was due to the inhibition of DNA repair. These findings suggest that endogenous miR-770-5p may function as an anti-oncogene and promote chemosensitivity in OVC, at least in part by downregulating ERCC2. miR-770-5p may therefore be a useful biomarker for predicting chemosensitivity to cisplatin in OVC patients and improve the selection of effective, more personalized, treatment strategies.

## INTRODUCTION

Ovarian cancer (OVC) is the leading cause of death due to gynecologic malignancies among women in developed countries. Epithelial ovarian cancer (EOC), which has a poor prognosis due to late diagnosis and high incidences of chemoresistance, accounts for approximately 90% of OVCs [1, 2]. The standard treatment protocol for the initial management of OVC is cytoreductive surgery, followed by primary chemotherapy with a platinum-based regimen [3, 4]. However, drug resistance is a major impediment to the successful treatment of OVC with chemotherapy. Chemoresistance often results in significant toxicities, such as declining bone marrow reserves, which delay the initiation of therapy with active chemo-agents and reduce quality of life [3]. Early prediction of OVC patient response to platinum-based chemotherapy and identification of the most effective agent based on primary tumor gene expression data may assist in optimizing the selection of personalized treatment regimens.

Platinum (cisplatin or carboplatin) is the first-line chemotherapeutic drug for OVC patients and a mainstay of standard treatment for advanced EOC [5, 6]. The major effect of cisplatin treatment involves DNA binding, the production of intrastrand (or interstrand) structural crosslinks, and the formation of DNA adducts [7–10]. Nucleotide damage repair and DNA repair capacity (DRC) play critical roles in the removal of cisplatin-induced lesions, leading to altered cell growth, differentiation, apoptosis, and carcinogenesis, and thus affecting response to chemotherapy and chemoresistance [7, 11].

Recent evidence indicates that microRNAs (miRNAs) play important roles in various pathways related to anticancer drug resistance, e.g., influencing the response to the conventional chemo-agents cisplatin and microtubule-targeting drugs [12–15]. However, whether and how miRNAs predict chemosensitivity and mediate drug resistance in OVC requires further investigation. In this study, we identified miRNAs that are differentially expressed in EOC patients who show complete responses (CR) or incomplete responses (IR) to primary platinum-based chemotherapy. We coupled this analysis with bio-functional experiments to determine how oncogenic-miRNA signaling pathways impact platinum-resistance. Our findings suggest that integrating miRNA expression profiles that predict platinum response, and understanding the mechanisms by which miRNAs affect chemoresistance, might improve the selection of personalized treatment plans for individual EOC patients.

## RESULTS

### Patient characteristics

We evaluated 86 serous EOC samples resected at the time of primary surgery from patients who subsequently received platinum-based primary therapy. All samples were collected at the Obstetrics and Gynecology Hospital, Dalian, China between July 2004 and November 2010. Fifty-two EOC patients showed a CR, and 34 showed an IR, to primary platinum-based therapy following surgery. Patient clinical characteristics are listed in Table 1.

**Table 1:** Clinicopathologic characteristics of ovarian cancer patients

Characteristic	Clinical Complete Responders (*n* = 52)	Clinical Incomplete Responders (*n* = 34)
Meanage,years	50.8	48
Stage, No. of patients		
I	3	2
II	8	7
III	41	21
IV	0	4
Grade, No. of patients		
1	3	2
2	28	14
3	21	18
Mean serum CA-125, m/mL		
Before platinum	993.2	1884.4
After platinum	12.1	205.6
Mean survival time, months	46	30
Mortality rate (%)	48.3	70

### miR-770-5p is downregulated in chemotherapy-resistant EOC patients

**Figure 1:**
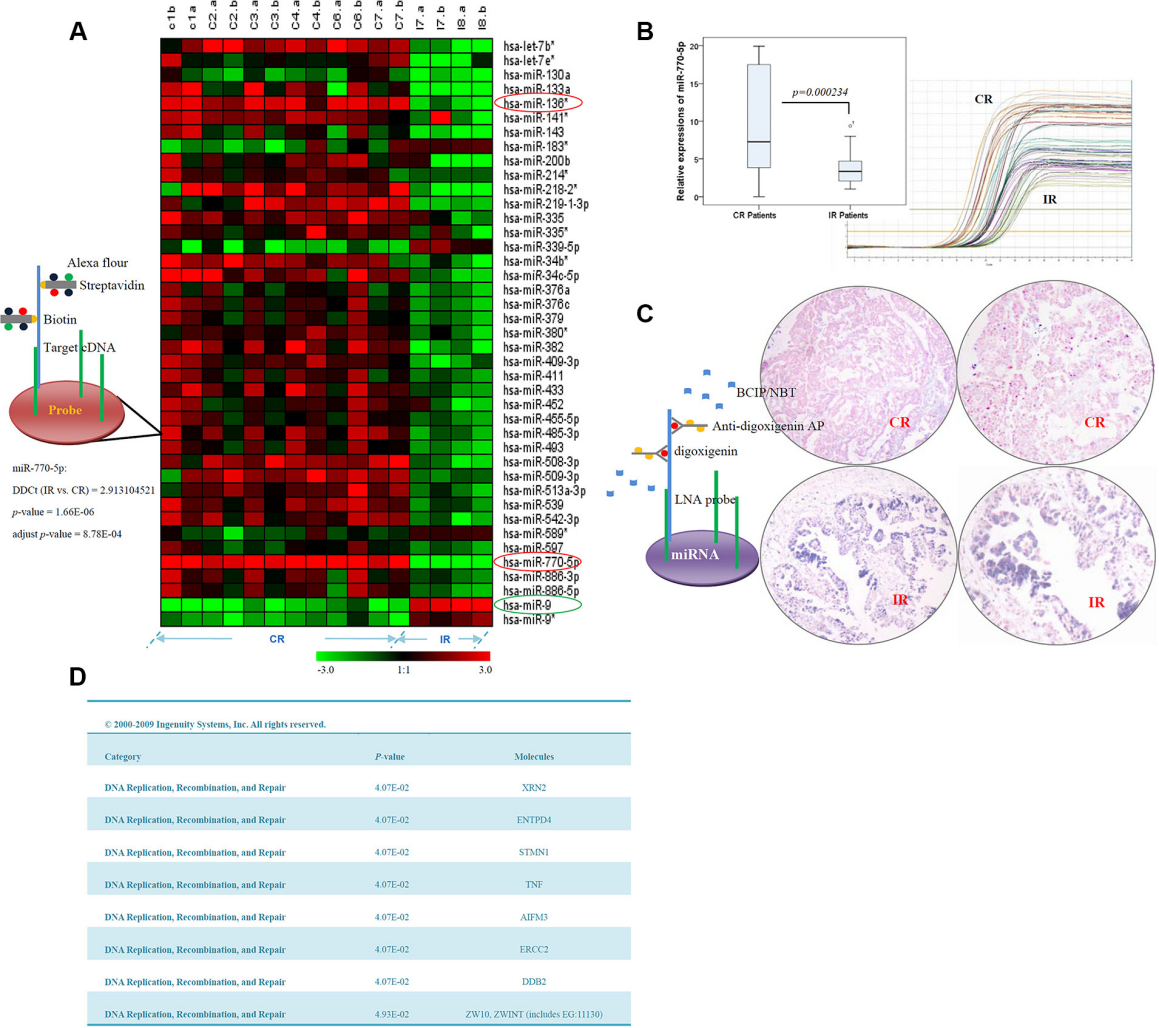
miR-770-5p expression is reduced in patients with chemotherapy-resistant EOC, and target predictions (**A**) Hierarchical clustering of miRNA microarray analysis in complete response (CR) and incomplete response (IR) ovarian cancer patients. Two miRNAs (miR-770-5p and hsa-miR-136*, red circle) were more highly expressed (*p* < 0.0001) in the CR group, and one (hsa-miR-9, green circle) was more highly expressed in the IR group. (**B**) Differential miR-770-5p expression in CR and IR patients. (**C**) Analysis of miR-770-5p expression in CR and IR sample sets using *in situ* hybridization. miR-770-5p expression was higher in CR than in IR samples (*p* < 0.001). (**D**) miRNA target prediction and top bio-functional analysis using Ingenuity Systems software. The predicted top nine targets were related to DNA replication, recombination, and repair.

To identify miRNA expression signatures associated with resistance to chemotherapy in EOC patients, patient specimens were initially analyzed by miRNA microarray, the results of which were validated with qRT-PCR and qualitative *in situ* hybridization (ISH). Of the 768 miRNAs analyzed in the microarray, levels of 39 differed at least 2-fold (*p* < 0.05) between CR and IR patients (*n* = 7 /group). Thirty-four (87.2%) of these 39 miRNAs were upregulated in the CR samples, and 5 (12.8%) were upregulated in the IR specimens (Figure 1A). Importantly, 2 of the 3 miRNAs with the most statistically significant differences (*p* < 0.0001) were upregulated in CR patients; the other was upregulated in IR patients. One of these three miRNAs, miR-770-5p, was upregulated 2.9-fold in the CR group versus the IR group (*p* = 1.66E–06, adjusted *p*-value = 8.78E–04) (Figure 1A and Supplementary Table S1). To confirm this finding, we examined miR-770-5p expression using qRT-PCR and ISH. As shown in Figure 1B–1C, miR-770-5p expression was lower in IR patients than in CR patients.

Target prediction was performed for miR-770-5p using three computational approaches: Ingenuity Systems (Redwood City CA, USA), MicroCosm Targets version 5, and miRBase. As cisplatin primarily functions through binding or/and crosslinking to DNA, targets were predicted by focusing on the “DNA Replication, Recombination, and Repair” area. The top nine candidate targets related to DNA replication and repair functions are listed in Figure 1D.

### Prediction of chemotherapy response and survival rate in EOC patients based on miR-770-5p expression

**Figure 2:**
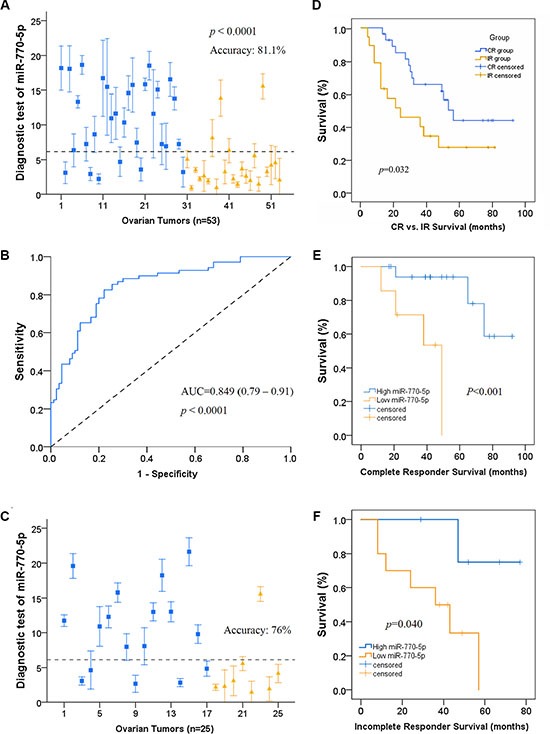
miR-770-5p levels predict response to platinum-based chemotherapy and survival in OVC patients (**A**) Leave-one-out cross prediction of cisplatin responders (blue: CR, yellow: IR), *n* = 53. a 6.128 in expression was the cutoff value having the highest Youden's index; accuracy, sensitivity, and specificity were 81.1%, 80%, and 83% respectively. (**B**) Receiver operating characteristic (ROC) curve of the cisplatin responders. A Mann-Whitney *U* test demonstrated that miR-770-5p expression distinguished IR patients from CR patients (*p* < 0.001). Mean AUC is 0.849 (0.79–0.91). (**C**) Confirmation that the 6.128 cutoff predicted cisplatin response prediction as indicated by ROC curve analysis. (**D**) In 47 randomly selected OVC patients, overall survival was higher in those with high miR-770-5p expression (CR = 28, IR = 19; *p* < 0.05). (**E**–**F**). Overall survival of CR and IR patients divided into high and low miR-770-5p subgroups. Kaplan-Meier survival analysis also indicated that low miR-770-5p expression was associated with shorter overall survival compared to the high expression group (*n* = 25 for CR and *n* = 15 for IR, *p* < 0.001 for CR group, *p* < 0.05 for IR group). AUC: area under the curve.

We then examined the relationship between miR-770-5p expression and primary chemo-responsiveness in patients in a retrospective study. A 53-sample training cohort was used to identify the miR-770-5p expression level that predicted clinical outcome. A 6.128-fold in expression served as the cutoff value for accurate prediction of response probability as determined by receiver operating characteristic (ROC) curve analysis (Figure 2A). Patient response to platinum-based therapy was accurately predicted in 43 of 53 patients for an overall accuracy of 81.1% (sensitivity, 80% and specificity, 83%) (Figure 2B). A Mann-Whitney U test for statistical significance (*p* < 0.001) confirmed that this predictor distinguished IR patients from CR patients. The mean AUC value (area under ROC curve) was 0.849.

To confirm the predictive value of miR-770-5p expression for primary chemosensitivity, we used miR-770-5p expression to predict primary chemoresistance in a separate group of patients before treatment with a platinum-based regimen. miR-770-5p expression accurately predicted primary chemotherapy response in this validation cohort (76% accuracy; Figure 2C). Kaplan-Meier survival analysis revealed that low miR-770-5p expression was associated with shorter overall survival in OVC patients compared to the high miR-770-5p expression group (Figure 2D–2F).

Taken together, these results indicate that miR-770-5p expression level may serve as a novel predictor and prognostic biomarker of OVC patient response to platinum-based chemotherapy and survival.

### Overexpression of miR-770-5p inhibits survival in cisplatin-chemoresistant cell lines

Based on the above results (Figure 1A–1C), we further confirmed the relationship between miR-770-5p expression and chemosensitivity in the human ovarian cancer cell lines OV2008 and A2780S and their cisplatin-resistant variants C13 and A2780CP, respectively. As indicated in Supplementary Figure S1, miR-770-5p expression was upregulated in OV2008 and A2780S cell lines compared to the cisplatin-resistant variants. These results agree with those from the clinical OVC patient specimens.

**Figure 3:**
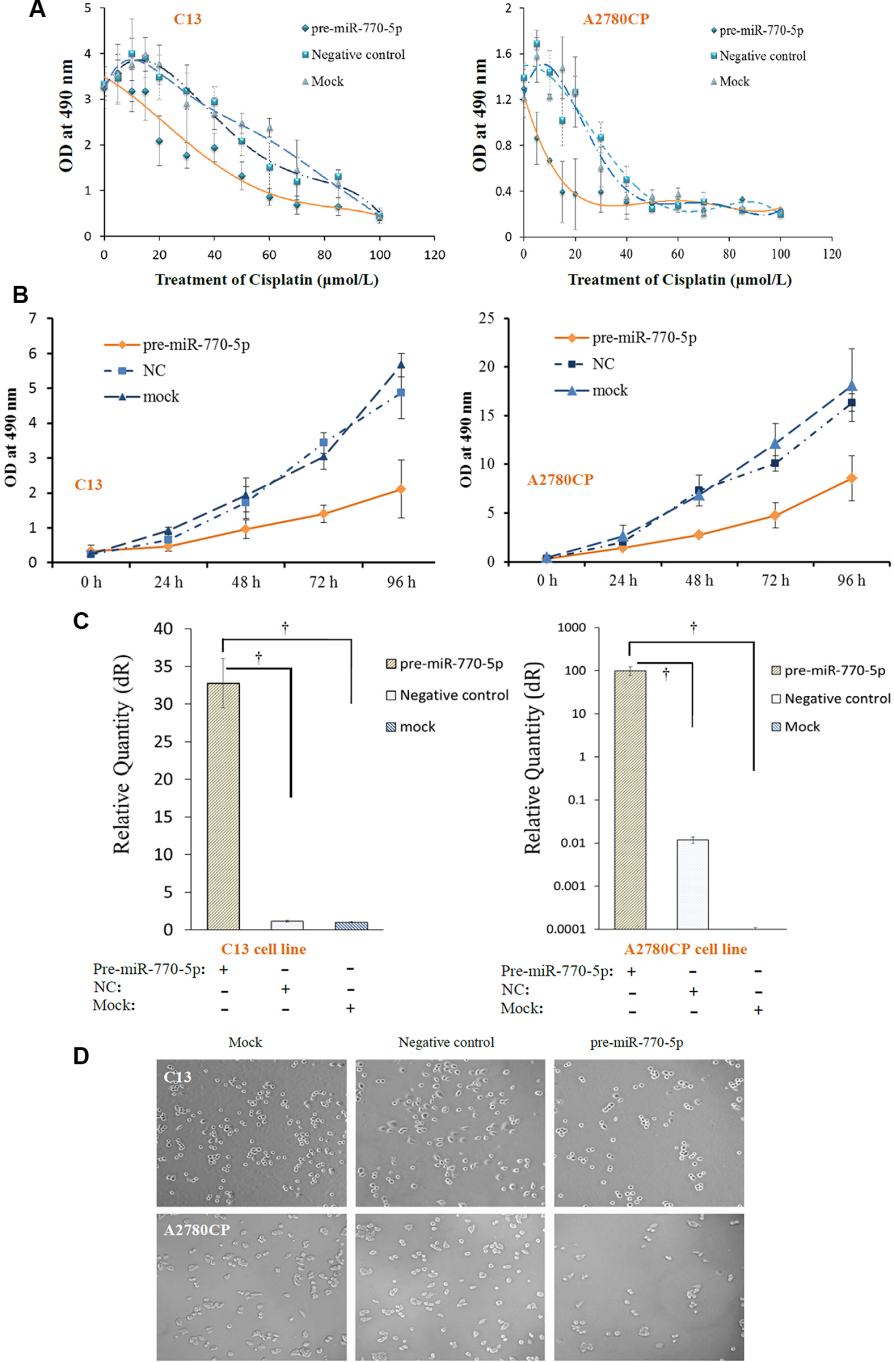
Overexpression of miR-770-5p inhibits survival of chemo-resistant cell lines after cisplatin treatment (**A**) Transfection with pre-miR-770-5p inhibited survival in C13 and A2780CP cells treated with various concentrations of cisplatin compared to mock and negative control groups (*n* = 10 for all groups). (**B**) Viability decreased in C13 and A2780CP cells after different lengths (0 to 96 h) of miR-770-5p transfection compared to mock and negative control groups (**C**) Real-time RT-PCR confirmed the success of pre-miR-770-5p transfection. (**D**) Pre-miR-770-5p, negative control, and mock transfected cell lines (C13 and A2780CP) were cultured in media containing cisplatin. Cell numbers were counted after 48 hours of treatment. ^†^, statistically significant difference in miR-770-5p expression compared to either negative control or mock-transfected cells (all *p* < 0.01).

We then investigated the effects of miR-770-5p overexpression on sensitivity to cisplatin chemotherapy in the resistant cell lines after treatment with various cisplatin concentrations or following various durations of miR-770-5p transfection *in vitro*. Transfection with pre-miR-770-5p reduced C13 and A2780CP cell viability relative to the mock and negative control groups (Figure 3A and 3B). C13 and A2780CP cell survival was also markedly decreased after pre-miR-770-5p transfection and 24 hours of cisplatin treatment (Figure 3D).

### ERCC2 is a potential target of miR-770-5p

**Figure 4:**
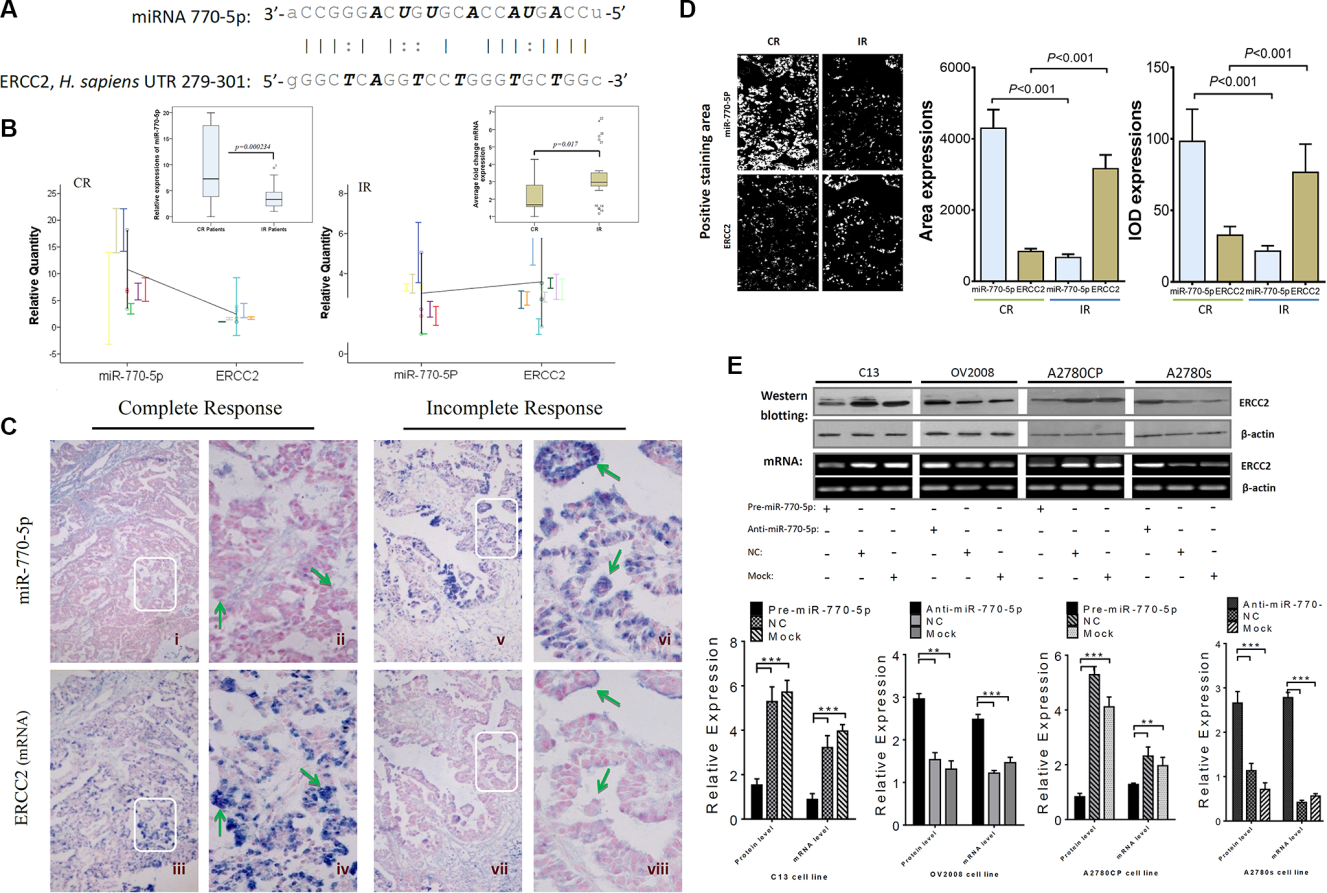
miR-770-5p inhibits ERCC2 *in vivo* and *in vitro* (**A**) Sequence alignment of human miR-770-5p with the 3'-UTR of ERCC2. (**B**) Differences in miR-770-5p (left panel) and ERCC2 (right panel) expression between the CR and IR groups. (**C**) Qualitative and quantitative examination of miR-770-5p and ERCC2 expression by ISH in FFPE sections from EOC patients. hsa-miR-770-5p was highly expressed in the tumor epithelium (green arrows in ii) of CR patients (i-iv), and ERCC2 expression was absent in the same tumor area (in iv). In contrast, IR tumors (v-viii) expressed low levels of hsa-miR-770-5p, but higher levels of ERCC2 (green arrows in viii). The areas in white boxes are shown enlarged on the right. (**D**) Quantitative analysis of miR-770-5p (miRNA) and ERCC2 (mRNA) expression in tumors conducted with Image-Pro 6.0 (positive areas: white color; IOD: integrated optical density) (**E**) Overexpression of miR-770-5p downregulates ERCC2 protein and mRNA levels in C13/A2780CP cells, and anti-miR-770-5p-treatment reversed this effect in OV2008/A2780S cells (all *p*< 0.05).

Nucleotide excision repair (NER) has been reported to play a role in regulating both chemoresistance and apoptosis [16]. ERCC2, one of the candidate target genes involved in NER, contains a putative region (nucleotides 279–301 in the human sequence 3'-UTR, NM_000400.2) that matches the seed sequence of hsa-miR-770-5p (Figure 4A). To determine whether ERCC2 is a target of miR-770-5p, quantitative and qualitative analysis at both the miRNA and mRNA levels were performed *in vivo* and *in vitro*.

First, miR-770-5p and ERCC2 expression were analyzed in CR and IR patients using qRT-PCR; miR-770-5p expression was negatively correlated with ERCC2 expression in both groups (Figure 4B). It is well-established that miRNAs suppress target gene expression primarily by inhibiting translation and/or induction of mRNA cleavage, and reciprocal expression patterns between an miRNA and the mRNA of its target gene can be detected in the same patient specimen. To confirm the miRNA-target relationship between miR-770-5p and ERCC2, miRNA and mRNA ISH was performed. Figure 4C shows the qualitative validation of miR-770-5p and its predicted target, ERCC2, in FFPE serial sections from same EOC patient. In the CR group, miR-770-5p was strongly expressed in the cancer epithelium, but ERCC2 expression was weak or undetectable in the same tumor tissues from the same patient. The same relationship between miR-770-5p and ERCC2 was also observed in IR patients. Quantitative analysis using Image-Pro 6.0 software confirmed an inverse relationship between miR-770-5p and ERCC2 mRNA expression in the CR and IR groups (Figure 4D).

To determine whether ERCC2 is also modulated by miR-770-5p *in vitro*, the cisplatin-sensitive parental cell lines (OV2008, A2780S) and their resistant variants (C13 and A2780CP) were transfected with anti-miR-770-5p or pre-miR-770-5p, respectively. Western blotting and semi-quantitative RT-PCR analyses revealed that miR-770-5p negatively regulated ERCC2 expression at both the mRNA and protein levels (Figure 4E).

### ERCC2 and miR-770-5p modulate sensitivity of ovarian cancer cells to cisplatin-induced apoptosis

**Figure 5:**
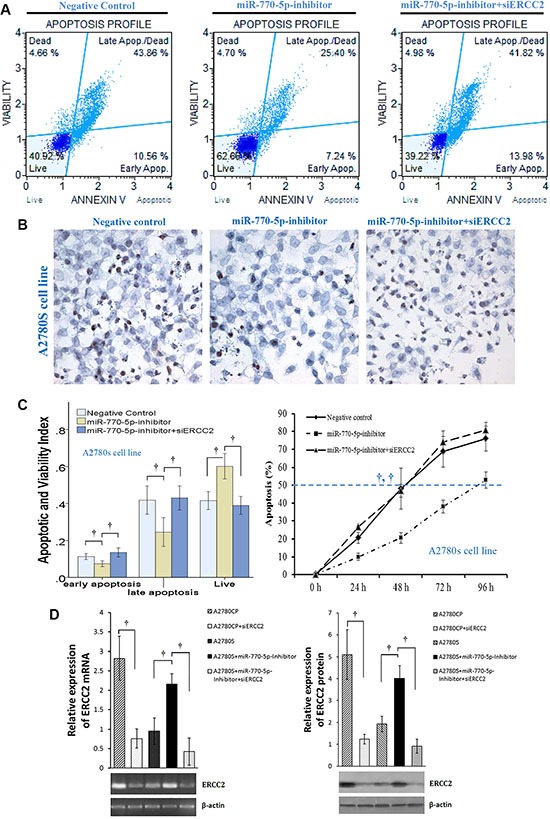
Effects of ERCC2 on miR-770-5p-modulated chemoresistance in ovarian cancer cells (**A**) Flow cytometric detection of apoptosis via Annexin V-FITC/PI staining in cisplatin-sensitive A2780S cells after treatment with cisplatin (20 μM) for 48 h after transfection with miR-770-5p inhibitor, negative control, or miR-770-5p inhibitor + siERCC2. (**B**) After transfection with miR770-5p inhibitor, A2780S cells were treated with cisplatin (40 μM) for 24 h. TUNEL staining indicated that apoptosis decreased after cisplatin treatment in A2780S cells transfected with miR-770-5p inhibitor; siRNA-mediated ERCC2 silencing reversed this effect. Original magnification: 400× (**C**) Analysis of flow cytometry results (left, *n* = 10/group) and TUNEL staining (right, *n* = 6/group). Statistical analyses of percent values and time to 50% apoptosis were conducted using Pearson's χ^2^ test and ANOVA, respectively. (**D**) RT-PCR and western blot analysis confirmed that siRNA inhibited the expression of ERCC2 mRNA and protein in A2780 cell lines. ^†^*p* < 0.001.

To determine how ERCC2 modulates chemosensitivity, we used siRNA to silence ERCC2 expression (siERCC2) in A2780S cells transfected with an miR-770-5p inhibitor. We then measured cisplatin-induced apoptosis in these cells with flow cytometry and TUNEL assays. A2780S cells became more resistant to cisplatin after transient transfection-mediated miR-770-5p silencing (Figure 5A–5C). Furthermore, silencing ERCC2 expression in A2780S cells treated with the miR-770-5p inhibitor restored chemosensitivity to cisplatin as indicated by both flow cytometry and TUNEL assays (Figure 5).

### Upregulation of miR-770-5p and downregulation of ERCC2 inhibits the repair of cisplatin-induced DNA-damage *in vitro*

**Figure 6:**
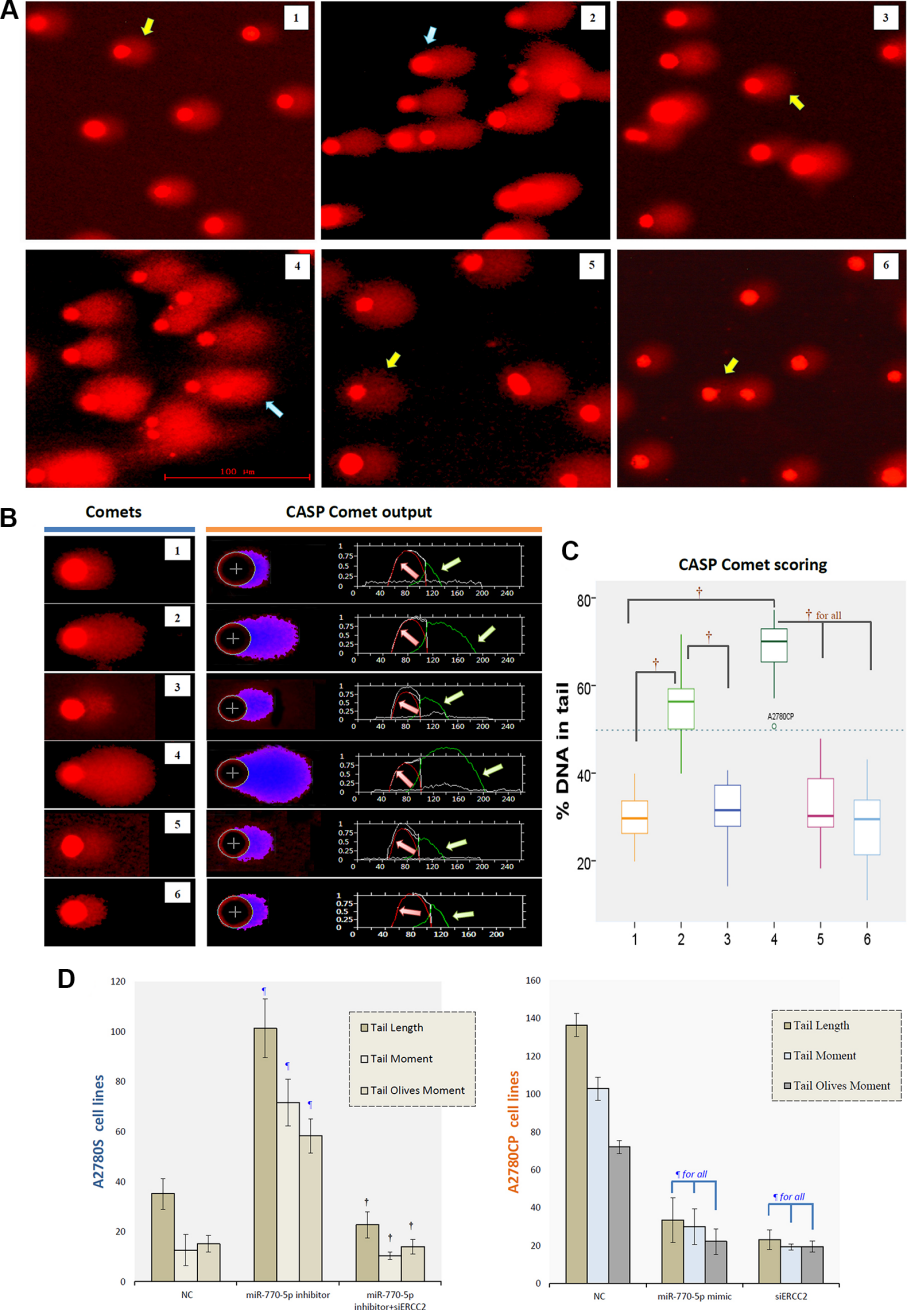
CASP comet analysis of alkaline comet assay images (**A**) Representative image of the modified COMET assay used to evaluate DNA damage after treatment with cisplatin. The blue arrow indicates a comet with a long tail, and yellow arrow a comet with a short tail. The scale bar is 100 μm. (**B**) Alkaline comet assay images for each group after cisplatin treatment are shown on the left, and CASP comet output and analysis are shown on the right. Red lines (indicated by red arrows) represent the head of comet, and green lines (indicated by green arrows) represent the tail of comet. (**C**) Amounts of DNA damage with and without cisplatin treatment in each group. ^†^*p* < 0.001. 1: A2780S NC, 2: A2780S miR-770-5p inhibitor, 3: A2780S miR-770-5p inhibitor + siERCC2, 4: A2780CP NC, 5: A2780CP miR-770-5p mimics, 6: A2780CP siERCC2. (**D**) Spontaneous and cisplatin-induced means ± S.D. for three additional replicates of the Comet assay after cisplatin treatment. ^¶^*p* < 0.001 compared to negative controls of the same cell type; ^†^*p* < 0.001 compared to the scrambled miR-770-5p or mimics group of the same cell type.

Cisplatin and other platinum-based cancer drugs destroy tumor cells by binding to DNA strands, interfering with DNA replication, and forming cisplatin-DNA adducts; chemosensitivity results from the inability to repair this DNA damage. Because the effects of miR-770-5p on DNA repair might explain its ability to modulate cisplatin-resistance in ovarian cancer, we examined the role of ERCC2 in this repair process. In order to quantify the DNA damage/repair process, we measured the percentage of DNA in comet tails; tail length (in μm), tail moment (TM), and tail olives moment (TOM) were measured in each gel (Figure 6A–6B). DNA damage was measured manually and evaluated with CASP version 1.2.3 beta2 (Figure 6C–6D). As expected, DNA damage repair-deficient A2780S cells had smaller tails than A2780CP cells. After transfection with the miR-770-5p inhibitor, A2780S DNA repair abilities increased, suggesting that these cells became more resistant to cisplatin. However, siRNA-mediated ERCC2 silencing reversed this increase. In addition, silencing ERCC2 inhibited DNA damage repair similarly to miR-770-5p upregulation in A2780CP cells (Figure 6). A representative image of the modified COMET assay used for data analysis is shown in Figure 5B. These results indicate that the effect of miR-770-5p on cisplatin-sensitivity was at least partly mediated by ERCC2.

## DISCUSSION

Platinum-based chemotherapy is the standard first-line treatment for advanced-stage EOC, and cisplatin is the drug used most often after primary surgery [3]. However, about 30–40% of patients who receive platinum-based chemotherapy experience disease progression or rapidly develop resistance to this non-targeted therapy [3, 17]. Therefore, early identification of platinum sensitivity would benefit platinum-resistant EOC patients, for whom other first-line therapies could be used [3, 17, 18–22].

Using miRNA microarrays, qRT-PCR, and ISH, we demonstrated that some miRNAs are differentially expressed in EOC patients who show CRs or IRs to primary chemotherapy. Preliminary experiments and comprehensive computational analysis identified miR-770-5p as a particularly relevant miRNA. miR-770-5p was downregulated in chemoresistant EOC patients, indicating that miRNAs are involved in the response to platinum-based regimens. To determine the impact of miR-770-5p on chemoresistance, we performed retrospective and prospective assessments of miR-770-5p as a predictor of primary chemosensitivity before and after patients received a platinum-based regimen. Unexpectedly, high miR-770-5p expression predicted chemoresistance with an accuracy of up to 81.1% in the retrospective and 76% in prospective assessments, with a 6.128-fold in expression serving as the cutoff value. This finding demonstrates that miR-770-5p expression immediately after primary EOC diagnosis can be used as a biomarker to assist in selecting the appropriate chemotherapy regimen before treatment begins, helping to avoid unnecessary toxicities and enhance quality of life. To confirm the association between miR-770-5p and chemo-resistance *in vitro*, we transiently transfected OVC cell lines with an miR-770-5p expression vector. Ectopic miR-770-5p expression increased survival and reduced apoptosis in ovarian cancer cells, suggesting that miR-770-5p also modulates therapeutic response.

To understand the mechanism by which miR-770-5p regulates chemoresistance in OVC, we identified possible target genes using computational approaches. One of the candidate targets was ERCC2, a gene in the NER system that is involved in DNA replication, recombination, and repair [7, 23–25]. Cisplatin activity is mediated through the formation of cisplatin-DNA adducts [26]. The NER system removes these adducts, which can lead to chemoresistance, and DRC thus plays a critical role in the response to chemotherapy and chemo-resistance [7, 11]; reduced DRC may increase the response to chemotherapies that damage cancer cell DNA. Here, we detected an inverse correlation between miR-770-5p and ERCC2 expression both in tumor specimens from OVC patients who received platinum-based chemotherapy and *in vitro*.

**Figure 7:**
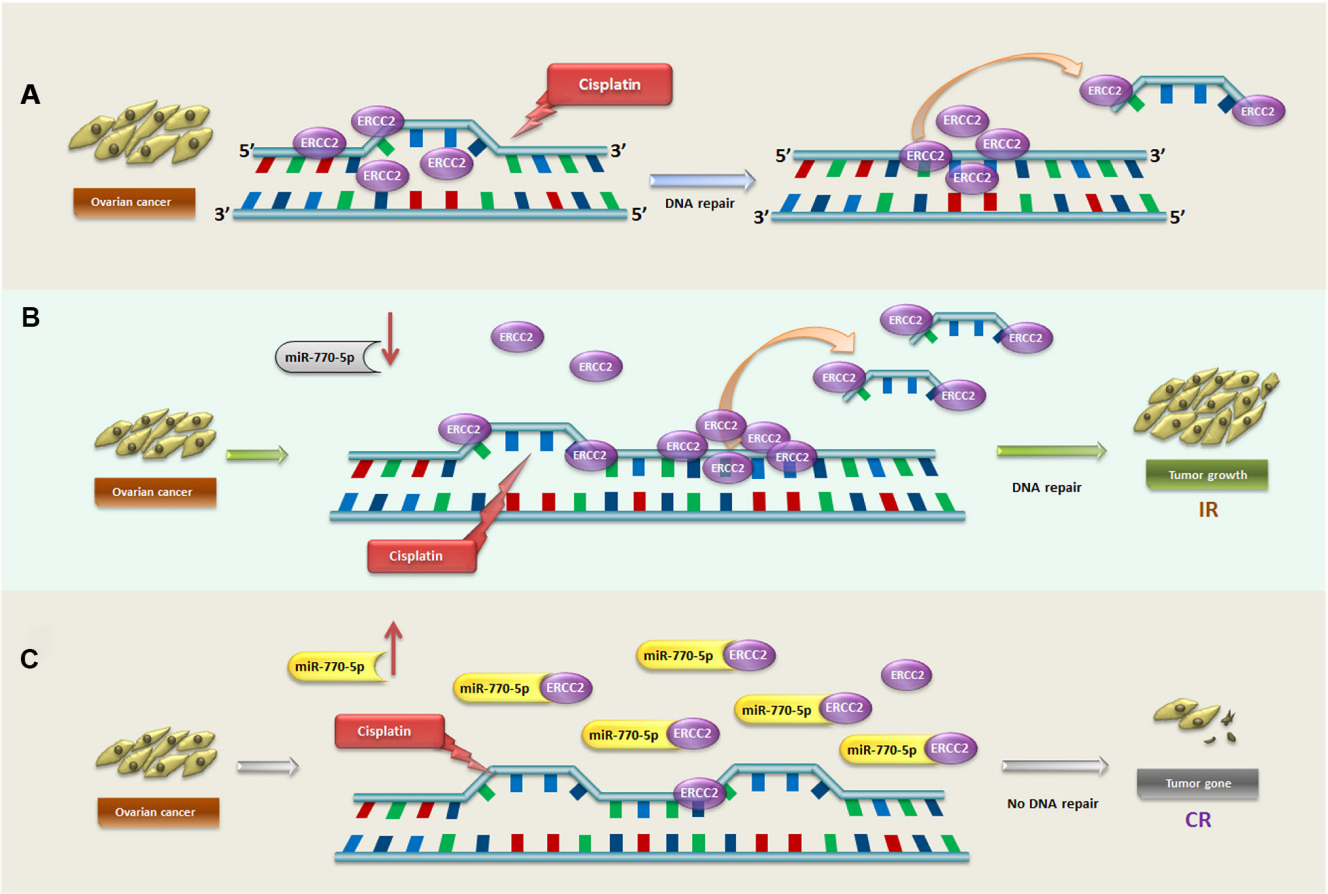
Schematic model depicting mediation of response to cisplatin-based primary chemotherapy by miR-770-5p and ERCC2 OVC patients (**A**) The general mechanism of ERCC2-dependent DNA damage repair after cisplatin treatment in OVC patients. After the DNA intra-strand is unwound and damaged by cisplatin, ERCC2 recognizes and repairs the DNA adducts. In OVC patients with IR to cisplatin, miR-770-5p is downregulated and does not suppress ERCC2 expression; ERCC2 is thus overexpressed in OVC cells and activates excision repair of DNA damaged by cisplatin, ultimately accelerating tumor cell growth (**B**) Conversely, in OVC patients with CR to cisplatin, miR-770-5p is upregulated and strongly suppresses ERCC2 expression in tumor cells; this reduces ERCC2-induced DNA damage repair, allowing cisplatin to damage DNA and ultimately activating tumor cell apoptosis (**C**) ERCC2 = excision repair cross-complementing rodent repair deficiency, group 2; IR = incomplete response; CR = complete response.

To determine how ERCC2 and miR-770-5p act together to modulate cisplatin-resistance, we used siRNAs to silence ERCC2 expression (siERCC2) in A2780S cells transfected with an miR-770-5p inhibitor. siRNA-mediated silencing of ERCC2 reversed the inhibition of apoptosis that resulted from miR-770-5p downregulation in A2780S cells. To investigate the mechanism underlying this effect, the comet assay, a sensitive and reliable method for studying DNA damage induced by platinum and other chemical agents, was used to assess DNA damage and DRC [27]. The results confirmed that the inhibition of apoptosis was due to increased DRC. Together, these findings suggest that miR-770-5p might directly target ERCC2, the downregulation of which increases cisplatin chemosensitivity in OVC. Thus, the anti-oncogenic and cisplatin chemosensitivity-enhancing effects of miR-770-5p in OVC might depend on its ability to downregulate ERCC2 expression (Figure 7).

Pharmacokinetic analyses of miRNAs are an innovative method for predicting treatment response and chemoresistance [12, 28]. In this study, we report that miR-770-5p enhances chemosensitivity at least in part by targeting ERCC2. Therefore, the miR-770-5p-ERCC2 interaction might be a useful biomarker for predicting chemosensitivity to cisplatin in OVC patients. Future examinations of miRNA expression profiles coupled with the expression of valid targets might improve predictions of sensitivity to primary chemotherapy and assist in the development of effective individualized cancer treatments.

## MATERIALS AND METHODS

### Patient samples and cell lines

We evaluated a total of 86 serous EOC samples resected at the time of primary surgery from patients who went on to receive platinum-based chemotherapy. The clinicopathologic characteristics of these patients are listed in Table 1. All tissue samples were collected at the Obstetrics and Gynecology Hospital, Dalian, China between July 2004 and November 2010. Two independent pathologists with no knowledge of the patients' clinical data reviewed the pathological specimens before they were formalin fixed and paraffin embedded (FFPE). Cases were classified according to the International Federation of Gynecology and Obstetrics (FIGO) staging system. Therapeutic response was evaluated as previously reported [29]. Medical records were examined by a single gynecologic oncologist and scored using standard criteria for patients with measurable disease based on WHO guidelines [29, 30]. A complete response (CR) was classified as a complete disappearance of all measurable and assessable disease or, in the absence of measurable lesions, by CA-125 levels < 30 U/mL measured by RIA or ELISA six months after treatment with platinum-based drugs. CA-125 response criteria were based on established guidelines [29, 31, 32]. Patients who exhibited only a partial response, had no response, or progressed during primary therapy were classified as having an incomplete response (IR). Fifty-two OVC patients showed a CR and 34 showed an IR after primary platinum-based therapy following the surgery. FFPE blocks were obtained after surgery.

*In vitro* experiments were performed with two different pairs of human OVC cell lines; each pair included one cisplatin-sensitive parental cell line (A2780S and OV2008) and its cisplatin-resistant variant (A2780CP and C13, respectively) [33, 34].

### Cell culture, transfection and treatment

Ovarian cancer cells were cultured in RPMI-1640 medium (Invitrogen, Burlington, ON) supplemented with 10% fetal bovine serum and maintained at 37°C with 5% CO_2_ as reported previously [33, 34]. All tissue culture reagents were obtained from Sigma-Aldrich (St. Louis, MO, USA). The miR-770-5p mimics and inhibitors were designed and chemically synthesized by Ambion (Cat#38422-01; Life Technologies Corporation, Denmark).

Lipofectamine 2000 (Invitrogen) was incubated with pre-miR-770-5p (miRNA mimic), anti-miR-770-5p (inhibitor), or scrambled negative controls (Ambion, mock) at a concentration of 90 nM and incubated in serum-free 1640 for 20 min before being added to cancer cells. Cells were incubated at 37°C for 4 h and then transferred to 10% FBS. Protein and RNA were harvested 48 h after transfection. For cisplatin treatment, cells were maintained in medium with the desired doses of cisplatin (Cat# P4394; Sigma, USA).

### miRNA microarray and data analysis

A microarray platform optimized for the analysis of a panel of 768 human miRNAs was used to analyze and compare miRNA expression patterns between EOC patients who showed a CR (*n* = 7) or an IR (*n* = 7) to platinum-based chemotherapy. Total RNA enriched for miRNAs was extracted from FFPE tissues using the Ambion mirVana microRNA isolation kit (Cat#AM1975; Ambion, USA). The quality of total RNA was assessed using an Agilent Bioanalyzer (Agilent Technologies, Santa Clara CA). Individual real-time quantitative polymerase chain reaction assays were performed in a TaqMan low-density array (TLDA; Applied Biosystems) by the Shannon McCormack Advanced Molecular Diagnostics Laboratory Research Services, Dana Farber Cancer Institute, Harvard Clinic and Translational Science Center. The normalized microarray data were analyzed using Statminer version 3.0 (Integromics™) software.

### miRNA target prediction and pathway analysis

miRNAs target prediction was conducted using three computational approaches: Ingenuity Systems (Redwood City CA, USA), MicroCosm Targets version 5 (http://www.ebi.ac.uk/enright-srv/microcosm/htdocs/targets/v5/), and miRBase (http://www.mirbase.org/). The target prediction algorithm used here is estimated to have a 20–30% false-positive rate. This level of false discovery is unlikely to affect the overall network findings, despite the large number of top predicted gene targets. Functional analysis of these predicted targets was performed to identify relevant biological pathways according to significant gene expression. To incorporate information from previous research on the identified miRNAs, the target networks of the relevant biological pathways, especially those related to cancer development, were predicted using commercially available software (Ingenuity Systems, Redwood City CA).

### Quantitative real-time PCR

Quantitative Real-Time PCR (qRT-PCR) was performed using the TaqMan MicroRNA Reverse Transcription Kit (Applied Biosystems, Foster City CA, USA) on an Agilent Technologies Stratagent Mx3000P (USA). A 500 ng mass of total RNA in 1 μL of RNase-free water was used in 20 μL of RT mix. The following primer pairs were used: has-miR-770-5p (ABI miRNA specific primers, ABI#002002), ERCC2 sense: 5'-CATGGCATACCAGAGAGCATATCC-3' and antisense: 5'-AGTTGAGCAACTTTCGAAGCTCTTC-3' (GenePharma Co., Ld, ShangHai, China). The products were detected with SYBR Green I, and relative miRNA or mRNA levels were calculated using the comparative Ct (cycle threshold, 2^-DDCt^) method with U6 and GAPDH as the endogenous controls, respectively. Samples from at least three independent experiments, each measured in duplicate, were analyzed, and the data are expressed as the mean ± SD.

### MTS cell viability assay

Cell survival was determined using the CellTiter 96^®^ AQueous Non-Radioactive Cell Proliferation Assay kit (Cat#P9625; Promega Co., USA). Briefly, cells were cultured in 96-well plates at a density of 1 × 10^3^/well for 48 h after transfection, and then treated with 0, 5, 10, 15, 20, 30, 40, 50, 60, 70, 85 or 100 μM cisplatin for 48 h. MTS/PMS solution composed of 20 μL each of a novel tetrazolium compound [3-(4,5-dimethylthiazol-2-yl)-5-(3-carboxymethoxyphenyl)-2-(4-sulfophenyl)-2H-tetrazolium, inner salt; MTS] and an electron coupling reagent (phenazine methosulfate; PMS) was added to one 96-well plate for a total volume of 100 μL with cultured cells followed by incubation at 37°C in a humidified, 5% CO_2_ atmosphere for 1 h. The absorbance at 490 nm was measured using an ELISA plate reader (PowerWavex 340, Bio-Tek Instruments Inc., Winooski, VT, USA). Each point represents the mean ± S.D. of triplicates. Responses to drug treatment were assessed by standardizing treatment groups to untreated controls.

### Cellular apoptosis assay with flow cytometry and TUNEL

Annexin V binding with propidium iodide (PI) staining was performed using the Muse^TM^ Annexin V εt Dead Cell kit (Merck KGaA, Darmstadt, Germany). Ovarian cancer cells were seeded at 1 × 10^5^ cells/mL in 6-well plates and incubated at 37°C in a humidified atmosphere with 5% CO_2_ for 48 h after transfection. C13 cells were then treated with 30 μM, OV2008 cells with 20 μM, and A2780CP and A2780S cells with 15 μM cisplatin for 48 hours, after which cells were dissociated using 0.05% EDTA-free trypsin and washed with cold PBS. Approximately 1 × 10^6^ cells were suspended in 100 μL of Annexin V incubation reagent. After incubation in the dark for 20 minutes at room temperature, cellular fluorescence was analyzed with a Muse^TM^ Cell Analyzer (EMD Millipore Co., Germany) within 30 minutes. Control tubes containing binding buffer only and cells treated with Annexin V alone or PI alone were initially used to calibrate the instrument. The nuclear morphology of apoptotic cells was determined at the same time using the Showed TUNEL *in situ* apoptosis detection kit (KeyGene Biotech Inc., China) following the manufacturer's instructions. Apoptotic cells (brown staining) were counted under a microscope.

### miRNA and RNA *in situ* hybridization (ISH)

All reagents were maintained in an RNase-free environment to minimize RNase contamination. ISH was performed on patient FFPE sections according to previously described protocols [33]. The probes used for ISH were hsa-miR-770-5p (Exiqon; Cat#38422-01) and human ERCC2 (an EcoRI-BamH1 fragment with antisense and sense digoxigenin-labeled riboprobes which were *in vitro*-transcribed from the full-length human ERCC2 coding sequence according to the Boehringer-Mannheim-Roche protocol). After removing the liquid mounting medium (LMM, Cat#19477; Aqueous Mounting Media, Quick-Mount 480l Daido Sangyo Co. LDT, Tokyo Japan), the transferred sections were treated with proteinase K (30 μg/mL, Amresco; Cat No: 1227B016) for 5 min, followed by three washes in PBS. The sections were incubated with pre-hybridization buffer (10 mL formamide, 5 mL 20× SSC, 2 mL 50× Denhardt's, 250 μL 20 mg/mL yeast RNA, 1000 μL 10 mg/mL salmon sperm DNA, 0.4 g blocking powder, and 1.75 mL DEPC-treated water) at 55°C for 1 h. Hybridization buffer containing the probes for has-miR-770-5p or ERCC2 was applied to each section and hybridized overnight at 55°C. On the second day, after washing with 2× SCC, the sections were incubated with anti-DIG-Fab-AP (Roche; Cat#11376621) for 2 h at room temperature. After washing, the sections were stained with BCIP (3.5 μL/mL)/NBT (4.5 μL/mL) solution (Amresco; Cat# 0885/0329) in a humidified chamber at room temperature in the dark overnight. Then, the sections were counterstained with nuclear fast red. After dehydration in ascending concentrations of ethanol and clearing in xylene, the sections were mounted in mounting medium. Positive controls and no-probe controls were included for each hybridization procedure.

### Reverse transcription PCR

Total RNA was prepared using Trizol reagent following manufacturer's instructions. Reverse transcription-polymerase chain reaction (RT-PCR) was conducted as previously described [34, 35]. Newly synthesized cDNA was amplified by PCR. The 20 μL reaction mixture contained 10 μL of cDNA template, 5 × 5 μL PCR Buffer, 0.25 μL of Taq polymerase, and 0.5 μL of primer mixtures. The following primers were used: for ERCC2 expression, sense: 5'-CATGGCATACCAGAGAGCATATCC-3' and antisense: 5'- AGTTGAGCAACTTTCGAAGCTCTTC-3' (product size, 110 bp); for GAPDH (housekeeping gene) expression, sense: 5'-CATGAGAAGTATGACAACAGCCT-3' and antisense: 5'-AGTCCTTCCACGATACCAAAGT-3' (product size, 113 bp). The RT-PCR cycle was as follows: RT at 42°C for 45 min, 85°C for 5 min; PCR at 94°C for 30 s, 60°C for 30 s, and 72°C for 1 min, followed by 40 cycles of amplification. PCR products were analyzed with 1.5% agarose gel electrophoresis in the presence of ethidium bromide for UV light transilluminator visualization.

### Western blot analysis

Harvested cells were lysed in lysis buffer (Beyotime Institute of Biotechnology, Shanghai, CN), and lysates were evaluated for protein concentration using the BCA method (Beyotime Institute of Biotechnology, Shanghai, CN). Proteins (20 μg) were separated on 10% SDS-PAGE gels and transferred to nitrocellulose membranes. Membranes were blocked in PBS containing 0.05% Tween 20 (TBST)-5% nonfat milk and incubated with primary antibody (rabbit anti-ERCC2, 1:500 and rabbit anti-β-actin, 1:1000; ProteinTech) in PBST-5% nonfat milk overnight at 4°C. After secondary antibody incubation, membranes were visualized with BeyoECL Plus A/B detection kit (Beyotime Institute of Biotechnology, Shanghai, CN).

### Comet assay

The Single Cell Gel Electrophoresis assay (also known as Comet Assay) is a sensitive technique for measuring DNA strand breaks in individual cells. This is a standard technique for evaluation of DNA biomonitoring, genotoxicity, and damage/repair [27, 36]. The assay was performed as described by Carlos et al. [34] using the DNA Damage Detection Kit (KeyGEN BioTECH, Inc, Nanjing, China) with some modifications. Cells were exposed to cisplatin for 1 h, washed, and incubated at 37°C with 5% CO_2_ for 48 hours. Next, the cells were trypsinized and resuspended in 1 mL culture medium. After that, 10 μL of cells (1 × 10^4^) were encapsulated in 75 μL 0.7% low-melting-point agarose at 37ºC. This mixture was layered onto slides precoated with 0.5% standard agarose and covered with a coverslip. The agarose was allowed to solidify for 10 min at 4ºC, and then the coverslip was gently removed. Then, the slides were immersed in a precooled lysis buffer (Cat# KGA240) for at least 90 min at 4ºC followed by incubation in an alkaline buffer (1 mM EDTA and 300 mM NaOH; pH >13) for 60 minutes at room temperature to allow for DNA unwinding and alkali-labile site expression. Electrophoresis was conducted in the same alkaline buffer for 30 minutes at 25V (0.86 V/cm) and 300 mA. After electrophoresis, the slides were washed at least three times with 0.4 mM Tris-HCl (pH = 7.5) prior to staining with propidium iodide (PI) (20 μL) (KeyGEN BioTECH, Inc, Nanjing, China) for 10 min. Comet tails were visualized using a fluorescence microscope with a FITC filter. In these experiments, cells with high amounts of cisplatin-induced cross-links have shorter tails compared to cells in which cross-links have been repaired effectively. This is because unrepaired platinum-induced DNA cross-links remain together after DNA degradation, resulting in larger DNA fragments [37, 38]. The tail length is proportional to the DNA repair. DNA was stained and the percentage of DNA in comet tails, tail length (in μm), tail moment (TM), and tail olives moment (TOM) were quantified to determine the extent of DNA damage/repair (CASP 1.2.3beta2 version, Krzysztof Konca, CaspLab.com).

### Statistical analysis

SPSS 17.0 (Chicago, IL, USA) was used for all quantitative analyses, except for microarray data. Data are expressed as arithmetic mean ± SD of the number (n) of experiments. Samples were analyzed with repeated measures analysis of variance, and differences in incidences were analyzed using one-way ANOVA. Differences in positive area and integrated optical density (IOD) in each field of the ×400 *in situ* hybridization photographs were determined using Image-Pro plus vision 6.0. Overall survival was defined as the time from initial cytoreductive surgery to the date of the last follow-up or death. Survival time courses were evaluated using the Kaplan-Meier method, and groups were compared using the log rank test. *p* < 0.05 was considered statistically significant.

## SUPPLEMENTARY MATERIAL FIGURE AND TABLE


