# Supplementary material for: MiR-770-5p inhibits cisplatin chemoresistance in human ovarian cancer by targeting ERCC2
Source: Oncotarget. 2016 Jul 20;7(33):53254–68. doi: 10.18632/oncotarget.10736 (PMC5288183; doi:10.18632/oncotarget.10736)
Supplement: Supplementary file 1 [file oncotarget-07-53254-s001.pdf]

MiR-770-5p inhibits cisplatin chemoresistance in human ovarian cancer by targeting ERCC2

Supplementary Materials

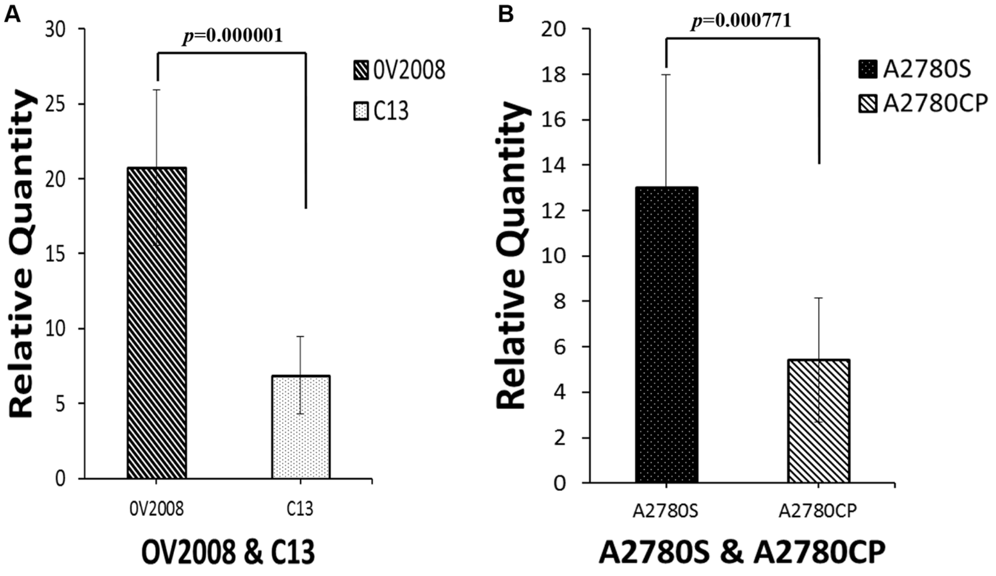

Supplementary Figure S1: In ovarian cancer cell lines, we also found that miR-770-5p expressions were all higher in cisplatin-sensitive parental cell lines (OV2008, A2780S) than their cisplatin-resistant variants (C13 and A2780CP), and *p*-values were 0.000001 and 0.000771, respectively (*n* = 10 for all).

Supplementary Table S1: Microarray data of miR-770-5p expression and statistical results between complete response (CR) and incomplete response (IR) ovarian cancer patients

miRNA ID	DDCt_IR-CR	*P*.Value_IR-CR	adj.*P*.Val_IR-CR
hsa-let-7b*	5.099748187	3.71E-03	0.024288619
hsa-let-7e*	2.645125438	3.20E-03	2.16E-02
hsa-miR-130a	1.727257438	5.61E-03	3.24E-02
hsa-miR-133a	4.288033521	2.94E-03	0.02044633
hsa-miR-136*	3.118522688	3.90E-06	8.78E-04
hsa-miR-141*	4.175896438	1.55E-03	1.41E-02
hsa-miR-143	3.190906021	6.26E-04	1.41E-02
hsa-miR-183*	−2.048991896	8.05E-03	0.042126017
hsa-miR-200b	5.331050104	5.12E-04	1.36E-02
hsa-miR-214*	1.578972354	5.02E-03	3.08E-02
hsa-miR-218-2*	7.706831854	1.39E-04	7.21E-03
hsa-miR-219-1-3p	6.655311021	1.56E-03	1.41E-02
hsa-miR-335	2.345812104	8.17E-04	1.41E-02
hsa-miR-335*	1.950726938	9.84E-03	4.71E-02
hsa-miR-339-5p	−3.030099479	2.58E-04	9.88E-03
hsa-miR-34b*	4.106150854	7.15E-03	3.92E-02
hsa-miR-34c-5p	3.746044354	9.25E-03	4.59E-02
hsa-miR-376a	1.894456521	2.44E-03	1.79E-02
hsa-miR-376c	2.368034188	1.98E-04	9.57E-03
hsa-miR-379	1.855945521	6.36E-03	3.55E-02
hsa-miR-380*	2.033712188	3.10E-04	9.98E-03
hsa-miR-382	3.431527688	4.05E-03	2.60E-02
hsa-miR-409-3p	6.827316854	8.47E-03	4.33E-02
hsa-miR-411	1.962364771	1.96E-03	1.47E-02
hsa-miR-433	2.398972688	1.35E-03	1.41E-02
hsa-miR-452	2.324147354	5.53E-03	3.22E-02
hsa-miR-455-5p	1.901040521	1.35E-03	1.41E-02
hsa-miR-485-3p	2.329316521	9.67E-03	4.66E-02
hsa-miR-493	2.507201521	2.21E-04	9.88E-03
hsa-miR-508-3p	5.114927354	3.00E-03	2.07E-02
hsa-miR-509-3p	4.157091438	5.18E-04	1.36E-02
hsa-miR-513a-3p	2.114513188	4.83E-03	3.05E-02
hsa-miR-539	2.069714854	8.38E-03	4.32E-02
hsa-miR-542-3p	4.008906437	2.58E-03	1.86E-02
hsa-miR-589*	−1.357795396	6.27E-03	3.53E-02
hsa-miR-597	1.464802438	5.12E-03	3.10E-02
hsa-miR-770-5p	2.913104521	1.66E-06	8.78E-04
hsa-miR-886-3p	1.862852021	7.47E-03	4.04E-02
hsa-miR-886-5p	2.218379688	1.74E-03	1.41E-02
hsa-miR-9	−4.274845896	8.06E-05	5.05E-03
hsa-miR-9*	−3.257400396	5.23E-04	1.36E-02

The assay was found that 41 miRNAs were statistically different ($p < 0.05$) between the groups, with 36 (87.8%) miRNAs higher in the complete response group and 5 miRNAs (12.2%, green word) higher in the incomplete response group. Three miRNAs exhibited even greater statistical significance ($p < 0.0001$, purple word for *p*-value) and of those, two were higher in the complete response group and one was higher in the incomplete response group. MiR-770-5p was the top differential miRNA of them (expression ratio of IR to CR = 2.9, $p = 1.66E–06$, adjust *p*-value = 8.78E–04.
